# Influence of Educational Level and Healthy Habits on the Prevalence of Diabesity in a Spanish Working Population

**DOI:** 10.3390/nu14194101

**Published:** 2022-10-02

**Authors:** José Ignacio Ramírez-Manent, Bárbara Altisench Jané, Matías Tomás Salvà, Sebastiana Arroyo Bote, Hilda María González San Miguel, Ángel Arturo López-González

**Affiliations:** 1 Faculty of Medicine, University Balearic Islands, 07009 Palma, Balearic Islands, Spain; 2 IDISBA, Balearic Islands Health Research Institute Foundation, 07004 Palma, Balearic Islands, Spain; 3 General Practitioner Department, Balearic Islands Health Service, 07003 Palma, Balearic Islands, Spain; 4 Investigation Group ADEMA SALUD IUNICS, 07003 Palma, Balearic Islands, Spain; 5 Faculty of Dentistry, University School ADEMA, 07009 Palma, Balearic Islands, Spain

**Keywords:** cardiovascular risk, obesity, diabesity

## Abstract

Background: Diabesity is a growing problem internationally. Taking into account the importance of physical activity and diet in its prevention and treatment, the objective of this study was to delve into the impact of healthy habits on diabesity. Methods: A descriptive, cross-sectional study was carried out in 386,924 Spanish adult workers. Obesity was determined according to eleven different formulas. Life habits were also valued; sociodemographic variables; and educational level; as well as analytical and clinical variables such as blood pressure and blood glucose levels. The association between the different variables was performed using the chi-square and the Student’s *t*-tests when the samples were independent. A multivariate analysis was performed using the multinomial logistic regression test by calculating the odds ratio and a 95% confidence interval. The Hosmer–Lemeshow goodness-of-fit test was also performed. Results: The overall prevalence of diabesity ranged between 0.3% (95% CI 0.3–0.4) when obesity was assessed according to the abdominal volume index and 8.3% (95% CI 8.2–8.4) when evaluated according to the CUN-BAE (Clínica Universitaria de Navarra Body Adiposity Estimator) formula. The prevalence of diabesity was also higher in workers with a non-heart-healthy diet and in those who did not exercise regularly. Conclusions: The most disadvantaged socioeconomic classes are those with the highest prevalence of diabesity. It is important to prioritise prevention in populations and communities with the most unfavourable social and environmental conditions to reduce the burden of diabesity.

## 1. Introduction

Diabesity is a new term to define type 2 diabetes associated with obesity. In recent decades, the increase in obesity has been followed by a parallel increase in the prevalence of diabetes mellitus [1,2]. Currently, diabesity has become a worldwide epidemic that constitutes a major public health problem. It is estimated that by 2025, more than 300 million people will have diabesity [3]. In Spain, the only study of the prevalence of diabesity that we have found was carried out by our group and it ranged between 2.6% and 5.8% people, depending on the formula used to measure obesity [4]. Both metabolic alterations level up the risk of presenting cardiovascular diseases [1,3,5], high blood pressure [1,5,6], and cerebrovascular accidents [1,5,7]; while diabetes is the main cause of blindness in adults [5,8], limb amputation [5,9], and kidney failure [5,10].

The expression diabesity, coined by Zimmet [11], has emerged from the combination of both terms (obesity and diabetes), and has been related to a decrease in both quality of life [12] and life expectancy [13], chronic stress [14], depression [15], and sleep disturbances [16].

Hypercaloric diets with a high intake of saturated fats together with low levels of physical activity cause significant concern in developed countries due to the increase in the prevalence of obesity, which should compel urgent measures to be taken, including both prevention and an early diagnosis in order to curb its progression. As obesity and diabetes are so linked, prevention and treatment must be carried out jointly.

In the prevention of diabesity, it is essential to modify lifestyle, for which the support of public institutions is necessary, acting in both directions: on the one hand, by educating the population to modify dietary habits, and on the other hand, by promoting physical activity on a regular basis [1,4,17,18]. In people with diabesity, it is indispensable to establish effective treatment based on weight loss by following the above parameters of a healthy diet and physical activity in order to reduce the aforementioned complications and, consequently, mortality [19]. A weight loss of between 10 and 15 kg can normalize blood glucose levels, with consequent health benefits [20]. However, it is well known that maintaining weight loss in people with diabetes is difficult to achieve, hence the most realistic goal would perhaps be to increase physical activity in order to control weight instead of fighting to lose it [21].

Taking into account the unanimity in the importance of physical activity and diet in the prevention and treatment of this clinical situation, the objective of this study was to delve into the impact of both healthy habits and other sociodemographic variables such as age, sex, and educational level on studies on the emergence or otherwise of diabesity in the Spanish population, on which there is very little published literature.

## 2. Materials and Methods

### 2.1. Type of Study and Sample

A descriptive cross-sectional study was carried out in a Spanish working population that attended periodic occupational health check-ups during the period between January 2020 and December 2021. The population included in the study was extracted from the anonymised database of the ADEMA-UIB university school (*Universitat de les Illes Balears*), which includes workers who have undergone medical examinations in the last 5 years at the national level (RD 688/2005 of 10 June and Law 31/95 on occupational risk prevention), with the approval of the Research Ethics Committee of the Balearic Islands. All activities were carried out following the ethical standards of the institutional research committee and the 2013 Declaration of Helsinki. All workers signed an informed consent to participate in the study.

Anthropometric, laboratory, and clinical variables were taken and recorded by the health personnel of the occupational health units of the companies that participated in the study after homogenising procedures.

### 2.2. Determination of Variables

The weight (in kilograms) and height (in centimetres) of the participants were obtained with a SECA 700 scale and a SECA 220 built-in height rod [22].

The waist and hip circumference were measured with a SECA 20 metric tape. For both measurements, the person stood upright with their feet together, their abdomen relaxed, and their upper limbs hanging. To measure the waist, the tape was held at the level of the last floating rib and parallel to the ground, and for the hip at the height of the buttocks.

Blood pressure was obtained after ten minutes of rest with the person in a sitting position, using an OMRON M3 automatic sphygmomanometer, by making three consecutive determinations and obtaining the average.

Laboratory tests were obtained by peripheral venepuncture after at least 12 h of fasting and then sent to the reference laboratories where they were analysed within 72 h. Automated enzymatic methods were used to determine glycaemia, cholesterol, and triglycerides (expressed in mg/dL). For the HDL, the dextran-sulphate technique was used (also expressed in mg/dL). The LDL was obtained by using the Friedewald formula (valid for triglyceride levels below 400 mg/dL).

LDL cholesterol = total cholesterol − HDL cholesterol − triglycerides/5

To classify glycaemia, the criteria of the American Diabetes Association (ADA) [23] were used, which establish hyperglycaemia from 125 mg/dL. Diabetics were considered to be people with a previous diagnosis, those with levels above 125 mg/dL, with glycated haemoglobin figures above 6.5%, or undergoing hypoglycaemic treatment.

### 2.3. Inclusion Criteria

1. Agree to participate in the study.
2. Work in one of the companies participating in the study.
3. Age between 18 and 69 years.
4. Have the variables in the database to calculate diabesity.

### 2.4. Exclusion Criteria

1. Decline to participate in the study.
2. Age under 18 or over 69.
3. Lack any variable to calculate diabesity scales.

### 2.5. Scales of Obesity

Different scales were used to determine obesity: waist/height ratio, waist/hip ratio, abdominal volume index (AVI) [24], body adiposity index (BAI) [25], body roundness index (BRI) [26], body shape index (ABSI) [27], relative fat mass (RFM) [28], ECORE-BF [29], CUN-BAE [30], METS-VF [31], and METS-IR [32] (Table 1).

### 2.6. Sociodemographic Variables and Tobacco

A person was considered a smoker when they had consumed at least 1 cigarette a day in the last 30 days or if they had stopped smoking less than 12 months before. A diet was considered healthy when the result of the values of the Mediterranean diet adherence questionnaire [33] was at least seven. Adequate physical exercise was considered when at least 150 min of moderate aerobic physical activity or 75 min of high-intensity physical activity were performed each week, or a combination of both.

Educational level was divided into three: no studies or primary studies, secondary studies (including secondary school or vocational training), and university studies (diplomas, graduate, and undergraduate studies).

The National Classification of Occupations of the year 2011 (CNO-11) was used, according to the proposal of the group of social determinants of the Spanish Society of Epidemiology to establish social class [34]. Three categories were established: class I (directors/managers, university professionals, athletes, and artists); II (intermediate occupations and self-employed workers without employees); and III (unskilled workers).

### 2.7. Statistical Analysis

Categorical type variables were described by frequency and percentage, and quantitative type variables by means and standard deviation (SD). To assess the association between the different variables, the chi-square test (with Fisher’s test if necessary) and Student’s *t*-test were used when samples were independent. Multivariate analysis was performed using the multinomial logistic regression test, calculating the odds ratio and 95% confidence intervals. The Hosmer–Lemeshow goodness-of-fit test was also performed.

The Pearson test was used to assess the correlation of the different obesity scales used. Cohen’s kappa coefficient was also used to assess the concordance of the different scales to diagnose diabesity.

Statistical calculations were performed with the SPSS 28.0 package, establishing a statistical significance level of *p* < 0.05.

### 2.8. Ethical Considerations and Aspects

The study was approved by the Clinical Research Ethics Committee of the Balearic Islands Health (Approval Code: IB 4383/20). All procedures were performed in accordance with the ethical standards of the institutional research committee and the 2013 Declaration of Helsinki. All participants signed written informed consent documents before participating in the study.

## 3. Results

### 3.1. Participants in the Study and Characteristics of Participants

**Figure 1:**
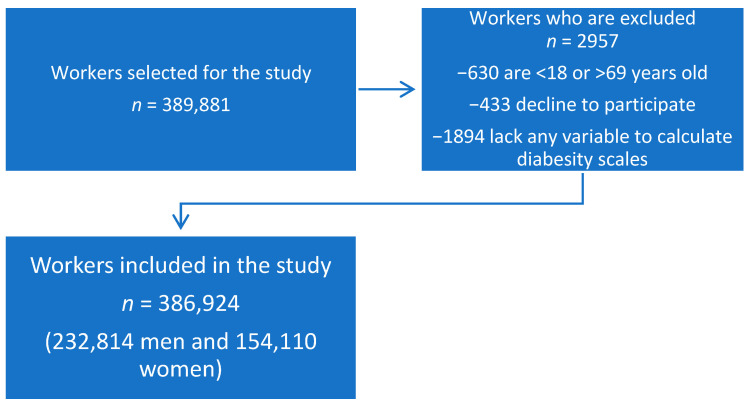
Flowchart of the study participants.

The study included 386,924 workers from different companies, notably: hospitality, construction, commerce, health, public administration, transportation, education, industry, and cleaning. The workers were from the autonomous communities of the Balearic Islands, Andalusia, the Canary Islands, the Valencian Community, Catalonia, Madrid, Castilla-La Mancha, Castile and León, and the Basque Country. The flowchart of the participants is presented in Figure 1.

In Table 2—where the anthropometric, clinical, laboratory, and sociodemographic data of the workers included in the study appear—we observe that the mean age was 39.6 years, and more than half were men (60.2%). All the analytical and clinical parameters presented worse values in men, except for LDL. Just one in three workers smoked. Almost half of people exercised regularly and a somewhat smaller percentage had a heart-healthy diet. The Autonomous Communities most represented in the study were Madrid, Catalonia, and Andalusia. Regarding the studies of the sample analysed, more than a half of the workers had primary studies, and most of them came from the tertiary sector.

### 3.2. Prevalence of Diabesity

The overall prevalence of diabesity ranged between 0.3% (95% CI 0.3–0.4) when obesity was assessed according to the abdominal volume index (AVI) and 8.3% (95% CI 8.2–8.4) when evaluated according to the CUN-BAE formula. In all of the formulas used to calculate the prevalence of diabesity, the result was much higher in men regardless of the formula used. An increase in diabesity was also found as age increased and with a lower level of education. The prevalence of diabesity was also higher in workers with a non-heart-healthy diet and those who did not exercise regularly. All these data were obtained regardless of the formula used to calculate diabesity. The complete data can be consulted in Table 3.

### 3.3. Multivariate Analysis

In the multivariate analysis (Table 4), it can be seen that being male increases the risk of presenting diabesity with all of the scales, being especially important if we apply BAI (OR 13.1; 95% CI 12.3–14.1) or METS-VF (OR 18.2; 95% CI 15.9–20.8). Age is also a factor that increases risk, especially on the METS-VF scale (OR 46.9; 95% CI 35.7–61.5, when comparing younger workers with older ones). Educational level is also a factor that influences the risk of developing diabesity, as seen with all of the scales. The two factors that show the most influence in increasing the risk of developing diabesity are a non-heart-healthy diet and not doing regular physical activity.

### 3.4. Correlation and Concordance between Different Scales

When applying Pearson’s coefficient (Table 5), we can see a correlation that ranges between moderate and strong in many of the scales that assess obesity, with very low statistical significance values *p* < 0.0001. The highest concordance levels between the different scales that assess diabesity using Cohen’s kappa index (k > 0.9) are found between CUN-BAE and ECORE-BF (0.998), waist/height index with BRI (0.993), METS-VF (0.925), and AVI (0.918) and between BRI and AVI (0.913).

## 4. Discussion

Our work shows the prevalence of diabesity in a working population by applying eleven different formulas with the intention of improving the effectiveness of prevention. The concept of diabesity arises from the coexistence in the same patient of two important and frequent pathologies: obesity and diabetes mellitus [4,10].

To determine obesity, most studies use the BMI or Quetelet index, a scale that uses height and weight without taking into account fundamental parameters such as lean mass and muscle mass, such that people who do a lot of physical exercise could be classified as overweight or with obesity as a result of their high percentage of muscle mass. In the same way, people with sarcopenia could be classified as normal weight despite having high levels of body fat. The BMI underestimates the prevalence of obesity by 50% when compared with direct fat measurement techniques, since its relationship with adiposity is influenced by age, sex, and race [35]. These variations make it advisable to use other methods to determine obesity, such as the evaluation of waist or hip perimeters, or the assessment or calculation of body fat levels [36,37].

In order to be able to act on a risk factor, it is necessary to know the underlying pathophysiological process. In both diabetes and obesity, their treatment is mainly based on changes in lifestyle [38,39,40], which makes a correct diagnosis of obesity essential since if this is not the case, it is possible that there will be no impact on the modification of unhealthy lifestyles in people with this risk factor, which would lead to treatment failure [40].

If we use the BMI as a formula for calculating diabesity, we find a much lower prevalence, in both men and women, than detected when applying other formulas that estimate body fat (ECORE-BF, CUN-BAE, and RFM). In the case of the RFM, it would only be influenced by sex; however, in the case of the ECORE-BF and the CUN-BAE, sex and age are included in the formula. Aging causes many changes in body composition: as a person gets older there is an increase in fat tissue, while muscle mass tends to decrease as well as body water content. Lipids infiltrate other tissues such as the liver, with hardly any changes in BMI values, however, these modifications have repercussions not only on health but also on methods to assess body composition [41,42].

Regardless of the formula used, the prevalence of diabesity is practically three times higher in men than in women, except for the formulas that include sex in their configuration (ECORE-BF, CUN-BAE, and RFM). This should be highlighted so that future studies take sex into account as a variable to be evaluated. In a previous study, we found that although the prevalence of diabesity is higher in men than in women, it is not so pronounced in formulas that include sex among their variables [4].

Aging also increases the prevalence of diabesity, which is basically a logical situation since, with increasing age, the prevalence of being overweight tends to increase and, therefore, more patients become diabetics, as aging and obesity are two risk factors for diabetes [43]. This fact is consistent with the data obtained in different national health surveys carried out between 1987 and 2012 in more than 150,000 people aged 16 and over [44].

In our work, we found diabesity levels inversely related to educational level, such that the highest percentages appear in people with only a primary education. We have found no studies that assess the relationship between educational level and diabesity directly, only between obesity (an important component of diabesity) and educational level. Thus, in the French ESTEBAN study of 2015, the prevalence of being overweight and obesity was higher among adults with the lowest educational level and among children whose caregiver did not have a school leaving certificate [45]. Similar results have been found in other studies [46,47].

Similarly, we have also found research studies that find a higher prevalence of diabetes in the population with a lower educational level, in this case more accentuated in women and younger individuals. These findings suggest that there are gender-based differences in lifestyle depending on the level of education and social class, that behave in a similar way in different geographical areas [48,49,50].

We know that social class generally has a good relationship with educational level. In several studies, also carried out by our group, the highest prevalence of diabesity occurs in people who belong to the most disadvantaged social groups [40], with a lower socioeconomic level [51]. Socioeconomic status is mainly defined by income, occupation, and educational level, which could reflect that these groups have less healthy lifestyles. Javed et al. found a prevalence of obesity between 50 and 70% higher in this group, in which psychological stress can also play an important role [52]. Several authors have found an important relationship between stress and obesity [53,54,55]

Regular physical exercise, especially moderate intensity aerobic training (minimum three days a week) decreases body mass index [56], visceral lipids, liver fat, and HbA1c in patients with diabesity [57]. This exercise intensity is sufficient to increase insulin sensitivity and lower plasma glucose levels [58,59,60]. In our work, physical exercise done on a regular basis has shown a very important effect on the prevalence of obesity, with an odds ratio ranging from 1.6, if the calculation is made with the ABSI formula, to 58.3, if evaluated according to the BMI, which confirms the importance of regular physical exercise in the prevention of diabesity. These data agree with those obtained by Abdelbasset et al. in an Egyptian population [56]. In a current study by Kirkpatrick et al., it was demonstrated in male rats that physical exercise could act on the orexinergic neurons of the lateral hypothalamus and interrupt the desire for high-fat foods [61].

A heart-healthy diet is the other factor that, in our work, was found to have a beneficial effect in reducing the prevalence of diabesity. This component is also highly influenced by socioeconomic level, in such a way that a low socioeconomic level is characterised by the consumption of foods with a high caloric component, such as sausages, fatty meats, whole milk, potatoes, pasta made with refined flours, sugars, sweets, and edible fats, and a low consumption of fruits, vegetables, and bread with wholemeal flour. These foods are cheaper, which enhances their purchase by this population and favours the development of obesity in this socioeconomic level and, consequently, diabetes [62,63,64]. Schusterbauer et al. also found an added difficulty in accessing new technologies which can help promote a more heart-healthy diet and physical exercise in patients with diabesity. However, these are more difficult for lower social classes and older patients to acquire and use [16].

Concordance between the different formulas used was assessed using the Pearson correlation coefficient, in which the results show a very high positive correlation between ECORE-BF and CUN-BAE, with *p* values < 0.0001.

The degree of concordance measured by the Kappa Cohen index for diabesity diagnosis is almost perfect between some of the formulas used, with a result of 0.993 between ICA and BRI; 0.925 between ICA and METS-VF; and 0.918 between ICA and AVI. All of them are very close to the unit. These results were expected since waist circumference is used as values in the four formulas, also introducing height among the three formulas that are closest.

Further, we found a Kappa Cohen index close to unity (0.998) between the CUN-BAE and ECORE-BF formulas, which was also to be expected since both formulas include age, sex, and BMI in their composition.

It is precisely these last two formulas that give us a higher prevalence of diabesity both globally and when separated by sex. It is known that both older people and women have a higher body fat percentage at the same BMI. There are multiple changes in body composition with aging: body fat increases and water content decreases, generally without changes in the BMI; thus, during aging the amount of fat increases and muscle mass or lean tissue decreases, and lipids enter other viscera such as the liver. These changes may affect procedures to assess body composition [41].

### Strengths and Limitations

The main limitation of our study is that it is a cross-sectional design, which does not allow causal relationships to be established, so no conclusions can be drawn about changes in anthropometric measurements over time. Secondly, the population in this study was ethnically homogeneous, since all of the patients in this study were Spanish, which could limit the generalisability of the findings. Furthermore, since it is a working population, it excludes groups of unemployed people and students. In addition, only patients who attended company medical check-ups were included.

One of the strengths of this study is the representativeness of the sample of the adult population in Spain: 386,924 workers, 154,110 women, and 232,814 men, as well as the use of eleven different formulas for the diagnosis of obesity.

## 5. Conclusions

The overall prevalence of diabesity in our population ranges from 0.3% when using the AVI to 8.3% when using the CUN-BAE formula, with a higher prevalence in men regardless of the formula used. The low sensitivity of the current BMI cut-off values could indicate that excess adiposity is being underdiagnosed in a significant part of the population, which may influence the adoption of necessary preventive measures to avoid its increase.

Our results have considerable connotations in the face of a growing international health problem such as diabesity, which increases morbidity and mortality and worsens the quality of life. The most disadvantaged socioeconomic classes are those with the highest prevalence of diabesity. It is important to prioritise prevention in populations and communities with the most unfavourable social and environmental conditions from the point of view of equity in health and to reduce the burden of diabesity, improve cardiovascular health and quality of life, and reduce the chronic pathologies associated with it.

## Figures and Tables

**Table 1:** Formulas to calculate obesity used in the study.

Formula	Values	Cut-Off Points for Obesity
Waist/height ratio waist/height circumference		>0.50
Waist/hip ratio waist circumference/hip circumference		≥0.85 women ≥0.95 men
AVI (abdominal Volume Index) =2 × waist circumference + 0.7 × (waist/hip)^2^/1000		>24.5
BAI (body adiposity index) =hip circumference/height 1.5–18		Women > 37.7 Men > 25.6
BRI (body roundness index) =364.2 − 365.5 × √1 − (waist circumference/2∏)^2^/(0.5 × height)^2^		>4.62
ABSI (body shape index) =waist circumference/BMI 1/3 × height 1/2)		>0.091
RFM (relative fat mass) Women 76 − 20 × (height/waist circumference) Men 64 − 20 × (height/waist circumference)		
		>32%
		>25%
ECORE-BF (Equation Córdoba for Estimation of Body Fat) =−97.102 + 0.123 × age + 11.9 × sex + 35.959 × LN BMI	Men = 0 Woman = 1	Women > 35% Men > 25%
CUN-BAE (Clínica Universitaria de Navarra Body Adiposity Estimator) =−44.988 + (0.503 × age) + (10.689 × gender) + (3.172 × BMI) − (0.026 × BMI^2^) + (0.181 × BMI × gender) − (0.02 × BMI × age) − (0.005 × BMI^2^ × sex) + (0.00021 × BMI^2^ × age)	Men = 0 Woman = 1	Women > 35% Men > 25%
BMI (body mass index) weight(kg)/height^2^ (meters)		≥30 kg/m^2^
METS-VF (metabolic score for visceral fat) =4.466 + 0.011 × LN(METS-IR)^3^ + 3.239 × LN(waist/height)^3^ + 0.319 × gender + 0.594 × LN (age)	Men = 1 Women = 0	>7.18
METS-IR = LN(2 × blood glucose + triglycerides) × BMI/LN(HDL)		

**Table 2:** Sociodemographic, anthropometric, clinical, and analytical characteristics of the sample.

	Total	Women	Men
	N = 386.924	N = 154.110 (39.8%)	N = 232.814 (60.2%)
Variables	Mean (SD)	Mean (SD)	Mean (SD)
Age (years)	39.6 (10.3)	39.2 (10.2)	39.8 (10.3)
Height (cm)	168.9 (9.3)	161.2 (6.6)	173.9 (7.0)
Weight (kg)	74.8 (15.6)	65.3 (13.2)	81.1 (13.9)
Abdominal perimeter (cm)	82.2 (11.0)	73.9 (7.9)	87.7 (9.1)
Hip circumference (cm)	98.9 (8.8)	97.2 (8.9)	100.1 (8.4)
Systolic blood pressure (mmHg)	120.4 (15.7)	114.4 (14.8)	124.4 (15.1)
Diastolic blood pressure (mmHg)	73.1 (10.9)	69.7 (10.3)	75.4 (10.6)
Cholesterol (mg/dL)	195.0 (37.9)	193.6 (36.4)	195.9 (38.9)
HDL (mg/dL)	52.1 (7.4)	53.7 (7.6)	51.0 (7.0)
LDL (mg/dL)	121.2 (37.4)	122.3 (37.0)	120.5 (37.6)
Triglycerides (mg/dL)	109.5 (76.3)	88.1 (46.2)	123.8 (88.0)
Glycemia (mg/dL)	86.5 (12.5)	84.1 (11.5)	88.1 (12.9)
variables	%	%	%
Age			
18–29 years	18.5	19.5	17.9
30–39 years	33.2	33.3	33.1
40–49 years	29.6	29.4	29.7
50–59 years	15.9	15.3	16.3
60–69 years	2.8	2.5	3.0
Educational level			
Primaries	57.4	51.8	61.2
Secondaries	36.7	40.7	34
University students	5.9	7.5	4.8
Smoking habit			
Nope	64.6	67.0	52.9
Yes	35.4	33.0	37.1
Regular physical exercise			
Nope	51.8	47.8	54.5
Yes	48.2	52.2	45.5
Heart-healthy diet			
Nope	54.9	48.6	59.0
Yes	45.1	51.4	41.0
Autonomous community			
Andalucia	14.7	14.0	15.2
Balearics Islands	6.2	5.8	6.5
Canary Islands	4.8	4.9	5.0
Castilla la Mancha	8.8	8.6	8.9
Castilla Leon	7.8	6.5	8.4
Catalonia	16.8	16.3	17.1
Valencian Community	10.9	11.3	10.4
Madrid	18.4	17.7	19.2
Basque Country	11.6	14.9	9.3
Productive sector			
Class I	4.1	3.9	4.3
Class II	23.1	6.3	31.5
Class III	72.8	89.8	64.2

SD: standard deviation.

**Table 3:** Prevalence of diabesity according to the formula used and for the different study variables.

					% (IC 95%)	Diabesity According to					
	ICA	WHR	AVI	BAI	BRI	ABSI	RFM	ECORE-BF	CUN-BAE	BMI	METS-VF
Global	6.3 (6.2–6.4)	1.8 (1.7–1.8)	0.3 (0.3–0.4)	4.7 (4.6–4.8)	2.2 (2.1–2.3)	2.0 (1.9–2.0)	7.9 (7.8–8.0)	8.2 (8.1–8.3)	8.3 (8.2–8.4)	3.8 (3.7–3.9)	1.8 (1.7–1.9)
Sex											
Women	2.1 (2.0–2.2)	0.6 (0.5–0.7)	0.1 (0.0–0.1)	0.6 (0.5–0.7)	0.5 (0.4–0.6)	0.6 (0.5–0.7)	3.6 (3.5–3.7)	4.9 (4.8–5.0)	5.1 (5.0–5.2)	2.3 (2.2–2.4)	0.1 (0.1–0.2)
Men	9.0 (8.9–9.1)	2.5 (2.4–2.5)	0.5 (0.4–0.6)	7.5 (7.4–7.6)	3.30 (3.2–3.4)	3.0 (2.9–3.1)	10.7 (10.6–10.8)	10.4 (10.3–10.5)	10.4 (10.3–10.5)	7.0 (6.9–7.1)	2.9 (2.8–3.0)
Age											
18–29 years	1.9 (1.7–2.1)	0.3 (0.2–0.5)	0.1 (0.0–0.1)	1.8 (1.7–1.9)	0.4 (0.3–0.5)	1.0 (0.9–1.1)	2.3 (2.2–2.4)	1.7 (1.6–1.8)	1.7 (1.6–1.8)	1.0 (0.9–1.1)	0.1 (0.1–0.2)
30–39 years	3.8 (3.7–3.9)	1.0 (0.9–1.1)	0.3 (0.2–0.4)	3.8 (3.7–3.9)	1.2 (1.1–1.3)	1.4 (1.3–1.5)	5.0 (4.9–5.1)	4.5 (4.4–4.6)	4.6 (4.5–4.7)	2.3 (2.2–2.4)	0.5 (0.4–0.6)
40–49 years	7.7 (7.6–7.8)	2.4 (2.3–2.5)	0.4 (0.3–0.5)	5.2 (5.1–5.3)	2.7 (2.6–2.8)	2.4 (2.3–2.5)	9.4 (9.3–9.5)	9.9 (9.8–10.0)	9.9 (9.8–10.0)	4.7 (4.6–4.8)	2.0 (1.9–2.1)
50–59 years	12.3 (12.1–12.5)	3.7 (3.5–3.9)	0.5 (0.3–0.7)	8.6 (8.4–8.8)	4.5 (4.3–4.7)	3.5 (3.3–3.7)	15.1 (14.9–15.3)	17.4 (17.2–17.6)	17.6 (17.4–17.8)	7.3 (7.1–7.5)	4.8 (4.6–5.0)
60–69 years	16.2 (15.5–17.0)	3.9 (3.2–4.6)	0.7 (0.3–1.2)	8.4 (8.0–8.8)	6.2 (5.8–6.6)	3.9 (3.5–4.3)	20.2 (19.8–20.6)	24.6 (24.2–25.0)	25.2 (24.8–25.6)	10.1 (9.7–10.5)	8.2 (7.8–8.6)
Educational level											
Primaries	7.3 (7.2–7.3)	2.0 (1.9–2.1)	0.4 (0.3–0.4)	5.7 (5.6–5.8)	2.5 (2.4–2.6)	2.3 (2.2–2.4)	9.2 (9.1–9.3)	9.5 (9.4–9.6)	9.5 (9.4–9.6)	4.3 (4.2–4.4)	2.1 (2.0–2.2)
Secondaries	5.1 (5.0–5.2)	1.5 (1.4–1.6)	0.3 (0.2–0.4)	3.7 (3.6–3.8)	1.8 (1.7–1.9)	1.7 (1.6–1.8)	6.4 (6.3–6.5)	6.8 (6.7–6.9)	6.8 (6.7–6.9)	3.1 (3.0–3.2)	1.5 (1.4–1.6)
University students	3.5 (3.0–4.0)	1.1 (0.7–1.6)	0.2 (0.1–0.4)	2.4 (1.9–2.9)	1.1 (0.7–1.6)	1.3 (0.9–1.7)	4.6 (4.2–5.0)	4.8 (4.4–5.2)	4.8 (4.4–5.2)	2.0 (1.6–2.4)	0.8 (0.4–1.2)
Regular physical exercise											
Nope	10.6 (10.5–10.6)	3.4 (3.3–3.5)	0.6 (0.5–0.6)	7.8 (7.7–7.9)	4.2 (4.1–4.3)	2.6 (2.5–2.7)	12.8 (12.7–12.9)	14.1 (14.0–14.2)	14.2 (14.1–14.3)	7.2 (7.1–7.3)	3.4 (3.3–3.5)
Yes	1.6 (1.5–1.7)	0.01 (0.01–0.02)	0.01 (0.0–0.02)	1.4 (1.3–1.5)	0.1 (0.0–0.2)	1.4 (1.3–1.5)	2.5 (2.4–2.6)	1.8 (1.7–1.9)	1.9 (1.8–2.0)	0.02 (0.01–0.03)	0.02 (0.01–0.03)
Healthy nutrition											
Nope	10.2 (10.1–10.3)	3.4 (3.3–3.5)	0.6 (0.5–0.7)	7.5 (7.4–7.6)	4.2 (4.1–4.3)	2.6 (2.5–2.7)	12.3 (12.2–12.4)	13.5 (13.4–13.6)	13.5 (13.4–13.6)	13.3 (13.2–13.4)	3.2 (3.1–3.3)
Yes	1.6 (1.5–1.7)	0.01 (0.01–0.02)	0.01 (0.0–0.02)	1.4 (1.3–1.5)	0.1 (0.0–0.2)	1.4 (1.3–1.5)	2.5 (2.4–2.6)	1.8 (1.7–1.9)	1.9 (1.8–2.0)	0.03 (0.02–0.04)	0.03 (0.02–0.04)

ICA = waist/height ratio. WHR = waist/hip ratio. AVI = abdominal volume index. BAI = body adiposity index. BRI = body roundness index. ABSI = body shape index. RFM = relative fat mass. ECORE-BF = Córdoba-body fat equation. CUN-BAE = Navarra University Clinic Body adiposity Estimator. BMI = body mass index. METS-VF = metabolic score for visceral fat.

**Table 4:** Multivariate model with the variables associated with diabesity (multinomial logistic regression).

						Diabesity According to					
	ICA	WHR	AVI	BAI	BRI	ABSI	RFM	ECORE-BF	CUN-BAE	IMC	METS-VF
	OR (IC 95%)	OR (IC 95%)	OR (IC 95%)	OR (IC 95%)	OR (IC 95%)	OR (IC 95%)	OR (IC 95%)	OR (IC 95%)	OR (IC 95%)	OR (IC 95%)	OR (IC 95%)
Women	1	1	1	1	1	1	1	1	1	1	1
Men	4.2 (4.0–4.4)	3.6 (3.3–3.8)	7.8 (6.2–9.7)	13.1 (12.3–14.1)	6.0 (5.5–6.4)	4.8 (4.5–5.1)	2.9 (2.8–3.0)	2.0 (2.0–2.1)	2.0 (1.9–2.0)	1.8 (1.7–1.8)	18.2 (15.9–20.8)
18–29 years	1	1	1	1	1	1	1	1	1	1	1
30–39 years	1.3 (1.2–1.4)	1.1 (1.0–1.2)	1.2 (1.1–1.3)	1.1 (1.0–1.2)	1.3 (1.2–1.4)	1.1 (1.0–1.2)	1.4 (1.3–1.4)	1.5 (1.4–1.6)	1.5 (1.5–1.6)	1.3 (1.2–1.4)	1.7 (1.6–1.9)
40–49 years	2.0 (1.9–2.1)	1.3 (1.1–1.4)	1.8 (1.6–2.0)	1.4 (1.3–1.5)	1.9 (1.7–2.1)	1.5 (1.4–1.7)	2.1 (2.0–2.2)	2.5 (2.4–2.6)	2.6 (2.5–2.7)	1.8 (1.7–1.9)	3.6 (3.3–3.9)
50–59 years	3.4 (3.2 (3.6)	2.2 (2.0–2.5)	2.1 (1.9–2.3)	1.6 (1.5–1.7)	3.3 (3.0–3.6)	2.4 (2.2–2.7)	3.4 (3.2–3.6)	4.7 (4.4–4.9)	4.8 (4.6–5.1)	2.8 (2.6–3.0)	11.0 (9.9–12.2)
60–69 years	5.5 (5.1–5.9)	4.7 (4.0–5.6)	4.8 (4.1–5.5)	2.7 (2.5–3.0)	6.3 (5.5–7.3)	3.3 (2.9–3.8)	6.1 (5.7–6.6)	10.3 (9.6–11.1)	10.5 (9.8–11.3)	4.4 (4.0–4.9)	46.9 (35.7–61.5)
University students	1	1	1	1	1	1	1	1	1	1	1
Secondaries	1.3 (1.2–1.3)	1.2 (1.1–1.2)	1.2 (1.0–1.3)	1.4 (1.3–1.4)	1.2 (1.2–1.3)	1.2 (1.2–1.3)	1.3 (1.3–1.4)	1.3 (1.2–1.3)	1.3 (1.2 (1.3)	1.2 (1.1–1.2)	1.2 (1.1–1.2)
Primaries	1.7 (1.6–1.8)	1.5 (1.3–1.7)	1.5 (1.4–1.6)	1.9 (1.7–2.0)	1.8 (1.6–2.0)	1.5 (1.3–1.7)	1.7 (1.6–1.8)	1.6 (1.5–1.8)	1.7 (1.6–1.8)	1.7 (1.5–1.8)	2.0 (1.7–2.3)
Yes physical exercise	1	1	1	1	1	1	1	1	1	1	1
Not physical exercise	3.5 (3.2–3.7)	41.7 (26.9–64.6)	13.8 (12.9–14.7)	3.2 (3.0–3.5)	22.2 (16.9–29.2)	1.6 (1.4–1.7)	2.8 (2.6–2.9)	3.8 (3.6–4.0)	3.8 (3.6–4.0)	58.3 (40.6–83.6)	33.4 (22.9–48.7)
Yes feeding	1	1	1	1	1	1	1	1	1	1	1
Not feeding	1.7 (1.6–1.8)	10.5 (6.8 (16.3)	10.5 (9.8–11.2)	1.5 (1.4–1.7)	4.8 (3.7–6.3)	1.1 (1.0–1.2)	1.6 (1.5–1.7)	1.9 (1.7–2.0)	1.8 (1.7–1.9)	19.6 (13.7–28.2)	4.0 (2.8–5.7)

OR = odds ratio. ICA = waist/height ratio. WHR = waist/hip ratio. AVI = abdominal volume index. BAI = body adiposity index. BRI = body roundness index. ABSI = body shape index. RFM = relative fat mass. ECORE-BF = Córdoba-body fat equation. CUN-BAE = Navarra University Clinic Body adiposity Estimator. BMI = body mass index. METS-VF = metabolic score for visceral fat.

**Table 5:** Correlation and consistency between the formulas used in the study.

				Pearson’s Coefficient							
	ICA	WHR	AVI	BAI	BRI	ABSI	RFM	ECORE-BF	CUN-BAE	BMI	METS-VF
ICA	1.000										
ICC	0.422	1.000									
AVI	0.091	0.235	1.000								
BAI	0.657	0.314	0.110	1.000							
BRI	0.499	0.689	0.252	0.437	1.000						
ABSI	0.364	0.473	0.148	0.187	0.463	1.000					
RFM	0.881	0.349	0.073	0.633	0.415	0.335	1.000				
ECORE-BF	0.772	0.324	0.069	0.612	0.392	0.210	0.840	1.000			
CUN-BAE	0.769	0.322	0.068	0.609	0.389	0.210	0.837	0.995	1.000		
IMC	0.623	0.369	0.138	0.602	0.501	0.125	0.594	0.608	0.604	1.000	
METS-VF	0.426	0.622	0.285	0.404	0.799	0.391	0.351	0.337	0.334	0.462	1.000
				**Cohen’s kappa index**							
ICA	1.000										
ICC	0.711	1.000									
AVI	0.918	0.713	1.000								
BAI	0.277	0.441	0.082	1.000							
BRI	0.993	0.689	0.913	0.306	1.000						
ABSI	0.431	0.720	0.482	0.436	0.415	1.000					
RFM	0.358	0.034	0.138	0.650	0.401	0.112	1.000				
ECORE-BF	0.244	0.272	0.086	0.751	0.283	0.591	0.805	1.000			
CUN-BAE	0.244	0.271	0.089	0.747	0.284	0.589	0.803	0.998	1.000		
IMC	0.684	0.184	0.633	0.557	0.687	0.325	0.376	0.673	0.672	1.000	
METS-VF	0.925	0.683	0.850	0.203	0.886	0.366	0.181	0.172	0.171	0.674	1.000

ICA = waist/height ratio. WHR = waist/hip ratio. AVI = abdominal volume index. BAI = body adiposity index. BRI = body roundness index. ABSI = body shape index. RFM = relative fat mass. ECORE-BF = Córdoba-body fat equation. CUN-BAE = Navarra University Clinic Body adiposity Estimator. BMI = body mass index. METS-VF = metabolic score for visceral fat.

## Data Availability

Data are available on request due to restrictions, e.g., privacy or ethical. Contact the corresponding author.

## References

[1] Bray G.A., Kim K.K., Wilding J.P.H., World Obesity Federation (2017). Obesity: A chronic relapsing progressive disease process. A position statement of the World Obesity Federation. Obes. Rev..

[2] Kheriji N., Boukhalfa W., Mahjoub F., Hechmi M., Dakhlaoui T., Mrad M., Hadj Salah Bahlous A., Ben Amor N., Jamoussi H., Kefi R. (2022). The Role of Dietary Intake in Type 2 Diabetes Mellitus: Importance of Macro and Micronutrients in Glucose Homeostasis. Nutrients.

[3] Ortega M.A., Fraile-Martínez O., Naya I., García-Honduvilla N., Álvarez-Mon M., Buján J., Asúnsolo Á., de la Torre B. (2020). Type 2 Diabetes Mellitus Associated with Obesity (Diabesity). The Central Role of Gut Microbiota and Its Translational Applications. Nutrients.

[4] López-González A.A., Ramírez Manent J.I., Vicente-Herrero M.T., García Ruiz E., Albaladejo Blanco M., López Safont N. (2022). Prevalence of diabesity in the Spanish working population: Influence of sociodemographic variables and tobacco consumption. An. Del Sist. Sanit. De Navar..

[5] Ng A.C.T., Delgado V., Borlaug B.A., Bax J.J. (2021). Diabesity: The combined burden of obesity and diabetes on heart disease and the role of imaging. Nat. Rev. Cardiol..

[6] El Khoury L., Chouillard E., Chahine E., Saikaly E., Debs T., Kassir R. (2018). Metabolic Surgery and Diabesity: A Systematic Review. Obes. Surg..

[7] Bhupathiraju S.N., Hu F.B. (2016). Epidemiology of Obesity and Diabetes and Their Cardiovascular Complications. Circ. Res..

[8] Wykoff C.C., Khurana R.N., Nguyen Q.D., Kelly S.P., Lum F., Hall R., Abbass I.M., Abolian A.M., Stoilov I., To T.M. (2021). Risk of Blindness Among Patients With Diabetes and Newly Diagnosed Diabetic Retinopathy. Diabetes Care.

[9] Kamitani F., Nishioka Y., Noda T., Myojin T., Kubo S., Higashino T., Okada S., Akai Y., Ishii H., Takahashi Y. (2021). Incidence of lower limb amputation in people with and without diabetes: A nationwide 5-year cohort study in Japan. BMJ Open.

[10] Braunwald E. (2019). Diabetes, heart failure, and renal dysfunction: The vicious circles. Prog. Cardiovasc. Dis..

[11] Pincock S. (2006). Paul Zimmet: Fighting the “diabesity” pandemic. Lancet.

[12] Horvath A., Leber B., Feldbacher N., Tripolt N., Rainer F., Blesl A., Trieb M., Marsche G., Sourij H., Stadlbauer V. (2020). Effects of a multispecies synbiotic on glucose metabolism, lipid marker, gut microbiome composition, gut permeability, and quality of life in diabesity: A randomized, double-blind, placebo-controlled pilot study. Eur. J. Nutr..

[13] Farag Y.M., Gaballa M.R. (2011). Diabesity: An overview of a rising epidemic. Nephrol. Dial. Transplant..

[14] Farzi A., Hassan A.M., Zenz G., Holzer P. (2019). Diabesity and mood disorders: Multiple links through the microbiota-gut-brain axis. Mol. Aspects Med..

[15] Bowen P.G., Lee L.T., Martin M.Y., Clay O.J. (2017). Depression and physical functioning among older Americans with diabesity: NHANES 2009–2010. J. Am. Assoc. Nurse Pract..

[16] Morselli L., Leproult R., Balbo M., Spiegel K. (2010). Role of sleep duration in the regulation of glucose metabolism and appetite. Best Pract. Res. Clin. Endocrinol. Metab..

[17] Schusterbauer V., Feitek D., Kastner P., Toplak H. (2018). Two-Stage Evaluation of a Telehealth Nutrition Management Service in Support of Diabesity Therapy. Stud. Health Technol. Inform..

[18] Castro E.A., Carraça E.V., Cupeiro R., López-Plaza B., Teixeira P.J., González-Lamuño D., Peinado A.B. (2020). The Effects of the Type of Exercise and Physical Activity on Eating Behavior and Body Composition in Overweight and Obese Subjects. Nutrients.

[19] Tilinca M.C., Tiuca R.A., Burlacu A., Varga A. (2021). A 2021 Update on the Use of Liraglutide in the Modern Treatment of ‘Diabesity’: A Narrative Review. Medicina.

[20] Lean M.E.J., Leslie W.S., Barnes A.C., Brosnahan N., Thom G., McCombie L., Peters C., Zhyzhneuskaya S., Al-Mrabeh A., Hollingsworth K.G. (2019). Durability of a primary care-led weight-management intervention for remission of type 2 diabetes: 2-year results of the DiRECT open-label, cluster-randomised trial. Lancet Diabetes Endocrinol..

[21] Nicklas B.J., Gaukstern J.E., Beavers K.M., Newman J.C., Leng X., Rejeski W.J. (2014). Self-monitoring of spontaneous physical activity and sedentary behavior to prevent weight regain in older adults. Obesity.

[22] https//www.seca.com/es_es.html.

[23] American Diabetes Association (2010). Diagnosis and Classification of Diabetes Mellitus. Diabetes Care.

[24] Guerrero-Romero F., Rodríguez-Morán M. (2003). Abdominal volume index. An anthropometry-based index for estimation of obesity is strongly related to impaired glucose tolerance and type 2 diabetes mellitus. Arch. Med. Res..

[25] Bennasar-Veny M., Lopez-Gonzalez A.A., Tauler P., Cespedes M.L., Vicente-Herrero T., Yañez A.M., Tomas-Salva M., Aguilo A. (2013). Body adiposity index and cardiovascular health risk factors in Caucasians: A comparison with the body mass index and others. PLoS ONE..

[26] Liu Y., Liu X., Guan H., Zhang S., Zhu Q., Fu X., Chen H., Tang S., Feng Y., Kuang J. (2021). Body Roundness Index Is a Superior Obesity Index in Predicting Diabetes Risk Among Hypertensive Patients: A Prospective Cohort Study in China. Front. Cardiovasc. Med..

[27] Krakauer N.Y., Krakauer J.C. (2018). Untangling Waist Circumference and Hip Circumference from Body Mass Index with a Body Shape Index, Hip Index, and Anthropometric Risk Indicator. Metab. Syndr. Relat. Disord..

[28] Segheto W., Marins J.C.B., Amorim P.R.D.S., Franco A.B., Almeida M.A., Alvarenga N.V.A., Lima L.M. (2021). Is relative fat mass a better indicator of high blood pressure levels when compared to other anthropometric indexes?. Nutr. Hosp..

[29] Molina-Luque R., Yañez A.M., Bennasar-Veny M., Romero-Saldaña M., Molina-Recio G., López-González Á.A. (2020). A Comparison of Equation Córdoba for Estimation of Body Fat (ECORE-BF) with Other Prediction Equations. Int. J. Environ. Res. Public Health.

[30] Costa A., Konieczna J., Reynés B., Martín M., Fiol M., Palou A., Romaguera D., Oliver P. (2021). CUN-BAE Index as a Screening Tool to Identify Increased Metabolic Risk in Apparently Healthy Normal-Weight Adults and Those with Obesity. J. Nutr..

[31] Kapoor N., Jiwanmall S.A., Nandyal M.B., Kattula D., Paravathareddy S., Paul T.V., Furler J., Oldenburg B., Thomas N. (2020). Metabolic Score for Visceral Fat (METS-VF) Estimation—A Novel Cost-Effective Obesity Indicator for Visceral Adipose Tissue Estimation. Diabetes Metab. Syndr. Obes..

[32] Bello-Chavolla O.Y., Almeda-Valdes P., Gómez-Velasco D., Viveros-Ruiz T., Cruz-Bautista I., Romo-Romo A., Sánchez-Lázaro D., Meza-Oviedo D., Vargas-Vazquez A., Campos O.A. (2018). METS-IR, a novel score to evaluate insulin sensitivity, is predictive of visceral adiposity and incident type 2 diabetes. Eur. J. Endocrinol..

[33] Riutord P., Riutord-Fe T., Riutord-Fe N., Arroyo S., López-González A.A., Ramirez-Manent J.I. (2022). Influence of physical activity and mediterranean diet on the values of different scales of overweight and obesity. Acad. J. Health Sci..

[34] Domingo-Salvany A., Bacigalupe A., Carrasco J.M., Espelt A., Ferrando J., Borrell C. (2013). Propuesta de clase social neoweberiana y neomarxista a partir de la Clasificación Nacional de Ocupaciones 2011. Gac. Sanit..

[35] Okorodudu D., Jumean M., Montori V., Romero-Corral A., Somers V., Erwin P., López Jiménez F. (2010). Rendimiento diagnóstico del índice de masa corporal para identificar la obesidad definida por la adiposidad corporal: Una revisión sistemática y un metanálisis. Int. J. Obes..

[36] Piché M.E., Tchernof A., Després J.P. (2020). Obesity Phenotypes, Diabetes, and Cardiovascular Diseases. Circ. Res..

[37] Wiechert M., Holzapfel C. (2021). Nutrition Concepts for the Treatment of Obesity in Adults. Nutrients.

[38] Wing R.R., Look AHEAD Research Group (2021). Does Lifestyle Intervention Improve Health of Adults with Overweight/Obesity and Type 2 Diabetes? Findings from the Look AHEAD Randomized Trial. Obesity.

[39] Moravcová K., Karbanová M., Bretschneider M.P., Sovová M., Ožana J., Sovová E. (2022). Comparing Digital Therapeutic Intervention with an Intensive Obesity Management Program: Randomized Controlled Trial. Nutrients.

[40] Faeh D., William J., Tappy L., Ravussin E., Bovet P. (2007). Prevalence, awareness and control of diabetes in the Seychelles and relationship with excess body weight. BMC Public Health.

[41] Baumgartner R.N., Heymsfield S.B., Lichtman S., Wang J., Pierson R.N. (1991). Body composition in elderly people: Effect of criterion estimates on predictive equations. Am. J. Clin. Nutr..

[42] Jungert A., Eichner G., Neuhäuser-Berthold M. (2020). Trajectories of Body Composition during Advanced Aging in Consideration of Diet and Physical Activity: A 20-Year Longitudinal Study. Nutrients.

[43] Wang M., Tan Y., Shi Y., Wang X., Liao Z., Wei P. (2020). Diabetes and Sarcopenic Obesity: Pathogenesis, Diagnosis, and Treatments. Front. Endocrinol..

[44] Toplak H., Leitner D.R., Harreiter J., Hoppichler F., Wascher T.C., Schindler K., Ludvik B. (2019). Diabesity“—Adipositas und Typ-2-Diabetes (Update 2019) [“Diabesity”-Obesity and type 2 diabetes (Update 2019)]. Wien Klin Wochenschr..

[45] Balicco A., Oleko A., Szego E., Boschat L., Deschamps V., Saoudi A., Zeghnoun A., Fillol C. (2017). Esteban design: A cross-sectional health survey about environment, biomonitoring, physical activity and nutrition (2014–2016). Toxicol. Anal. Et Clin..

[46] Do W.L., Bullard K.M., Stein A.D., Ali M.K., Narayan K.M.V., Siegel K.R. (2020). Consumption of Foods Derived from Subsidized Crops Remains Associated with Cardiometabolic Risk: An Update on the Evidence Using the National Health and Nutrition Examination Survey 2009–2014. Nutrients.

[47] Jimenez-Mora M.A., Nieves-Barreto L.D., Montaño-Rodríguez A., Betancourt-Villamizar E.C., Mendivil C.O. (2020). Association of Overweight, Obesity and Abdominal Obesity with Socioeconomic Status and Educational Level in Colombia. Diabetes Metab. Syndr. Obes..

[48] Bartolini L., Caranci N., Gnavi R., Di Girolamo C. (2020). Educational inequalities in the prevalence and outcomes of diabetes in the Emilian Longitudinal Study. Nutr. Metab. Cardiovasc. Dis..

[49] Abdullah A., Liew S.M., Salim H., Ng C.J., Chinna K. (2019). Prevalence of limited health literacy among patients with type 2 diabetes mellitus: A systematic review. PLoS ONE.

[50] Wu H., Bragg F., Yang L., Du H., Guo Y., Jackson C.A., Zhu S., Yu C., Luk A.O.Y., Chan J.C.N. (2019). Sex differences in the association between socioeconomic status and diabetes prevalence and incidence in China: Cross-sectional and prospective studies of 0.5 million adults. Diabetologia.

[51] Volaco A., Cavalcanti A.M., Filho R.P., Précoma D.B. (2018). Socioeconomic Status: The Missing Link Between Obesity and Diabetes Mellitus?. Curr. Diabetes Rev..

[52] Javed Z., Valero-Elizondo J., Maqsood M.H., Mahajan S., Taha M.B., Patel K.V., Sharma G., Hagan K., Blaha M.J., Blankstein R. (2022). Social determinants of health and obesity: Findings from a national study of US adults. Obesity.

[53] Bremner J.D., Moazzami K., Wittbrodt M.T., Nye J.A., Lima B.B., Gillespie C.F., Rapaport M.H., Pearce B.D., Shah A.J., Vaccarino V. (2020). Diet, Stress and Mental Health. Nutrients.

[54] Correia J.C., Locatelli L., Hafner C., Pataky Z., Golay A. (2021). Rôle du stress dans l’obésité [The role of stress in obesity]. Rev. Med. Suisse.

[55] Tomiyama A.J. (2019). Stress and Obesity. Annu. Rev. Psychol..

[56] Viana R.B., Naves J.P.A., Coswig V.S., de Lira C.A.B., Steele J., Fisher J.P., Gentil P. (2019). Is interval training the magic bullet for fat loss? A systematic review and meta-analysis comparing moderate-intensity continuous training with high-intensity interval training (HIIT). Br. J. Sports Med..

[57] Abdelbasset W.K., Badr N.M., Elsayed S.H. (2014). Outcomes of resisted exercise on serum liver transaminases in hepatic patients with diabesity. Med. J. Cairo. Univ..

[58] AbdelBasset W.K., Elsayed S.H., Nambi G., Alrawaili S., Elnegamy T.E., Khalil M.A., Tantawy S.A., Soliman G.S., Ibrahim A.A., Kamel D.M. (2020). Effect of Moderate-Intensity Aerobic Exercise on Hepatic Fat Content and Visceral Lipids in Hepatic Patients with Diabesity: A Single-Blinded Randomised Controlled Trial. Evid. Based. Complement Altern. Med..

[59] Ryan B.J., Schleh M.W., Ahn C., Ludzki A.C., Gillen J.B., Varshney P., Van Pelt D.W., Pitchford L.M., Chenevert T.L., Gioscia-Ryan R.A. (2020). Moderate-Intensity Exercise and High-Intensity Interval Training Affect Insulin Sensitivity Similarly in Obese Adults. J. Clin. Endocrinol. Metab..

[60] Myers J., Kokkinos P., Nyelin E. (2019). Physical Activity, Cardiorespiratory Fitness, and the Metabolic Syndrome. Nutrients.

[61] Kirkpatrick G.E., Dingess P.M., Aadland J.A., Brown T.E. (2022). Acute high-intensity interval exercise attenuates incubation of craving for foods high in fat. Obesity.

[62] Passos C.M.D., Maia E.G., Levy R.B., Martins A.P.B., Claro R.M. (2020). Association between the price of ultra-processed foods and obesity in Brazil. Nutr. Metab. Cardiovasc. Dis..

[63] Zhang Z., Kahn H.S., Jackson S.L., Steele E.M., Gillespie C., Yang Q. (2022). Associations between ultra- or minimally processed food intake and three adiposity indicators among US adults: NHANES 2011 to 2016. Obesity.

[64] de Mestral C., Chatelan A., Marques-Vidal P., Stringhini S., Bochud M. (2019). The Contribution of Diet Quality to Socioeconomic Inequalities in Obesity: A Population-based Study of Swiss Adults. Nutrients.

